# Multiple carcinosarcomas of the esophagus and stomach

**DOI:** 10.3892/ol.2012.1095

**Published:** 2012-12-28

**Authors:** FENG XU, WEN-BIN ZOU, XIAO-PING LI, YUE-MEI XU, XU-FEI QI, LIANG-HAO HU, ZHAO-SHEN LI, DING-KANG YAO

**Affiliations:** 1 Department of Gastroenterology, Yinzhou People’s Hospital, Ningbo, Zhejiang 315040;; 2 Department of Gastroenterology, Changhai Hospital, The Second Military Medical University, Shanghai 200433;; 3 Department of Gastroenterology, Changzheng Hospital, The Second Military Medical University, Shanghai 200003, P.R. China

**Keywords:** carcinosarcoma, upper gastrointestinal tract, immunohistochemistry

## Abstract

Carcinosarcoma is an uncommon biphasic malignant neoplasm consisting of both carcinomatous and sarcomatous components. We report a case of an 84-year-old male with multiple carcinosarcomas occurring in the esophagus and stomach. Endoscopically, a bulky pedunculated polypoid lesion was observed in the middle of the esophagus and a huge discoid lesion in the lesser curvature. The patient received esophageal endoscopic mucosal resection, and the specimen measured 4×2.5×1.5 cm. Microscopically, the esophageal tumor consisted of several polymorphic spindle cells mixed with squamous cells, while the gastric biopsies revealed carcinomatous cells with evident abnormal karyokinesis and polymorphous spindle cells. Immunohistochemically, the resected tumor stained positively for the epithelial markers, epithelial membrane antigen (EMA) and cytokeratin 19 (CK 19), and the mesenchymal markers, smooth muscle actin (SMA) and vimentin. The gastric lesion stained positively for CK AE1/AE3, actin and vimentin, but was negative for EMA. Both lesions were positive for neuron specific enolase (NSE), demonstrating neuroendocrine differentiation. The patient succumbed seven months after being discharged from hospital. To our knowledge, this is the first case in the literature that describes multiple carcinosarcomas arising from the esophagus and stomach. A review of the available literature is also presented.

## Introduction

Carcinosarcoma, also termed sarcomatoid carcinoma, pseudosarcoma or spindle cell carcinoma, is an unusual biphasic malignant neoplasm consisting of both carcinomatous and sarcomatous components. It is mainly characterized by a large, bulky and polypoid mass. Carcinosarcoma usually occurs in such diverse locations as the uterus, breast, thyroid, lung and upper gastrointestinal system. In this study, we describe a rare case of multiple carcinosarcomas (MCS) that occurred in the esophagus and stomach. To the best of our knowledge, this is the first report of MCS arising from both these regions. The study was approved by the Ethics Committee of Changhai Hospital, Shanghai, China. The patient provided written informed consent and accepted the therapy.

## Case report

**Figure 1:**
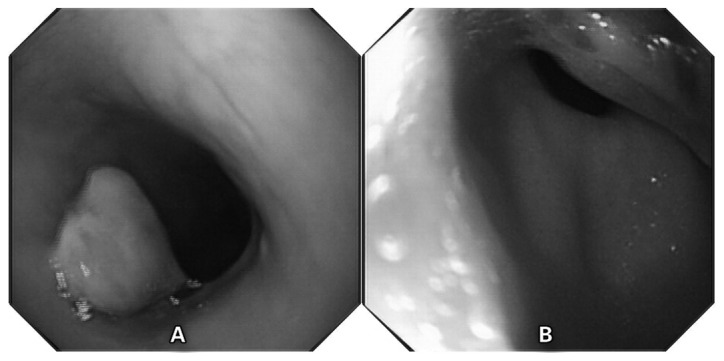
The first esophagogastroduodenoscopy. (A) In the esophagus, a pedunculated and lobulated polypoid lesion with erosive surface, measuring 12 mm in diameter and located 30 cm from the incisors is revealed. (B) In the gastric antrum, a huge mass with a hard texture accompanying the gastric cavity deformation is visible.

**Figure 2:**
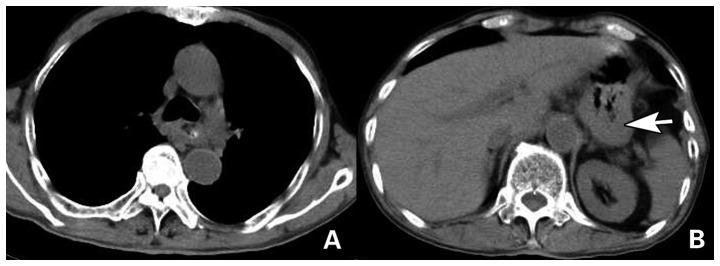
Computed tomography (CT). (A) Pectoral CT reveals a large, heterogeneous tumorous formation obturating the esophageal lumen, with no enlarged lymph node observed, located in the same plane as the lower tracheal bifurcation. The value of CT is ∼78 Hu. (B) The abdominal CT reveals the nodular increased thickness of the gastric antral wall and the narrowing gastric cavity.

**Figure 3:**
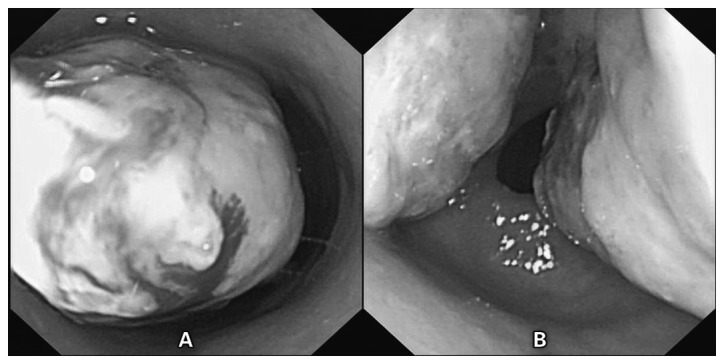
The second esophagogastroduodenoscopy. (A) In the esophagus, a bulky pedunculated polypoid lesion with a clear and easily bleeding surface, accompanying the narrowing lumen, is evident. (B) In the gastric antrum, a huge discoid lesion with a ‘dirty’ surface and a clear edge, accompanying the markedly deformed gastric cavity, is revealed.

**Figure 4:**
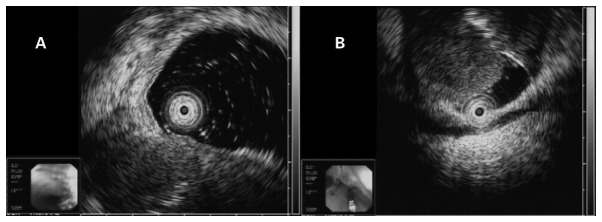
Endoscopic ultrasound. (A) In the esophagus, a hypoechoic mass with an unknown origin is revealed. (B) In the gastric corpus, a hypoechoic mass is evident.

**Figure 5:**
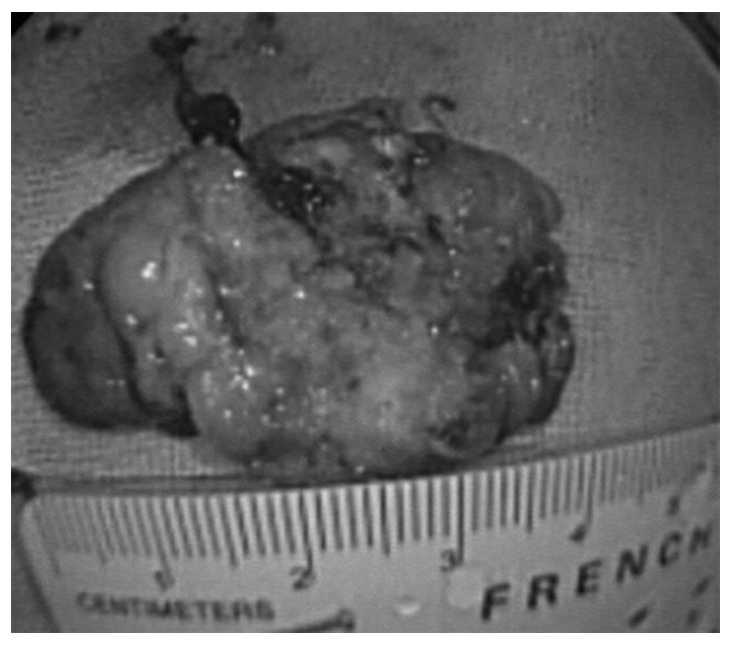
The resected tumor is 4×2.5×1.5 cm in size and contains blood clots.

**Figure 6:**
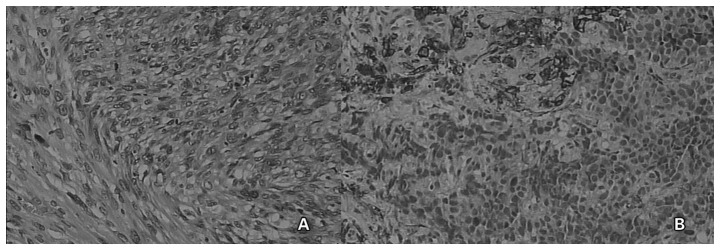
Microscopic findings. (A) The esophageal tumor consists of numerous spindle cells that are observed to be polymorphic, mixed with a number of squamous cells (hematoxylin and eosin; magnification, ×200). (B) The gastric biopsies reveal carcinomatous cells with evidently abnormal karyokinesis and polymorphic spindle cells (hematoxylin and eosin; magnification, ×200).

**Figure 7:**
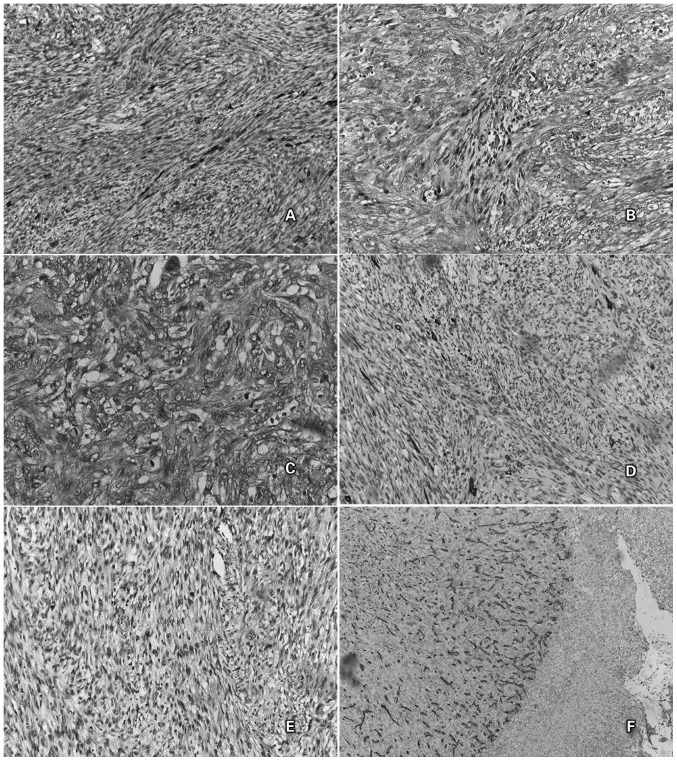
Immunohistochemical findings for the esophageal tumor. The staining results are as follows: (A) Strongly positive for EMA (×200); (B) strongly positive for SMA (×200); (C) strongly positive for vimentin (×400); (D) weakly positive for CK 19 (×200); (E) weakly positive for NSE (×200); (F) positive for CD 34, but only in the vascular endothelial cells (×100).

**Figure 8:**
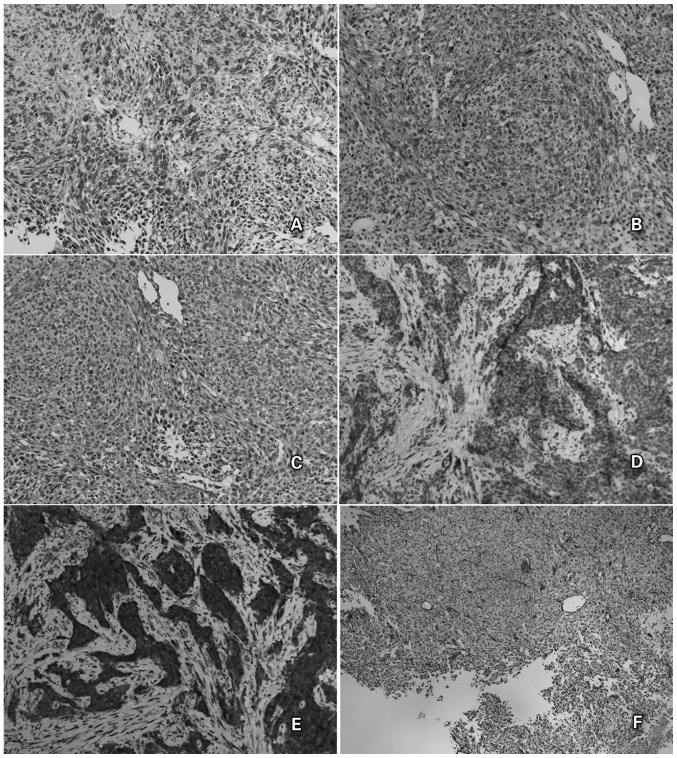
Immunohistochemical findings for the gastric biopsy. The staining results are as follows: (A) Strongly positive for CK AE1/AE3 (×200); (B) strongly positive for actin (×200); (C) weakly positive for vimentin (×200); (D) negative for EMA (×200); (E) positive for NSE (×200); (F) positive for CD 34, but only in the vascular endothelial cells (×100).

In April 2010, an 84-year-old male presented with recurrent epigastric pain for 9 months and melena for 5 months. The initial admission (4 months ago) revealed that the patient had severe anemia [hemoglobin, 5.5 g/dl; mean capuscular volume (MCV), 82.1 fl) with marked pallor. The patient had a long history of smoking exceeding 50 pack years (1000 cigarette years). The first esophagogastroduodenoscopy (EGD) revealed a pedunculated polypoid lesion in the esophagus and an elevated huge mass in the lesser curvature (Fig. 1). Both biopsies of the esophageal and gastric lesions revealed poorly differentiated squamous cell carcinoma, and the patient was negative for *Helicobacter pylori*. Without surgical indications, the patient received 4 cycles of chemotherapy using folinic acid, fluorouracil and oxaliplatin (FOLFOX). One week ago, the patient was re-admitted due to dysphagia. A computed tomography (CT) examination demonstrated a heterogenous tumorous formation obturating the esophageal lumen without enlarged lymph nodes in the thoracic region, while nodular thickening of the gastric antral wall and narrowing of the gastric cavity in the upper abdomen were observed (Fig. 2). Compared with the first EGD, the second revealed that the two lesions in the esophagus and stomach had evidently increased in size (Fig. 3). Endoscopic ultrasound (EUS) independently demonstrated a hypoechoic mass both in the esophagus and the gastric corpus (Fig. 4). To relieve the symptom of regurgitation, we performed a palliative esophageal endoscopic polypoid tumor resection (specimen size, 4×2.5×1.5 cm; Fig. 5) without bleeding and perforation. Microscopically, the resected tumor consisted of polymorphic spindle cells mixed with a number of squamous cells, while the gastric biopsies revealed carcinomatous cells with polymorphous spindle cells (Fig. 6). Immunohistochemically, the resected tumor stained positively for epithelial markers of epithelial membrane antigen (EMA) and cytokeratin 19 (CK 19), and mesenchymal markers of smooth muscle actin (SMA) and vimentin (Fig. 7). In the gastric lesion, staining was positive for CK AE1/AE3, actin and vimentin, but negative for EMA (Fig. 8). The aforementioned findings confirmed the diagnosis of multiple carcinosarcomas. Notably, both lesions stained positively for neuron specific enolase (NSE), demonstrating neuroendocrine differentiation (Fig. 7E and Fig. 8E), and positively for CD 34 in the vascular endothelial cells (Fig. 7F and Fig. 8F). The patient was discharged from hospital with normal food intake, and succumbed 7 months later.

## Discussion

In 1865, Virchow named the rare malignant neoplasm consisting of carcinomatous and sarcomatous components as ‘carcinosarcoma’ (1). Since then, it has also been referred to as sarcomatoid carcinoma, pseudosarcoma or spindle cell carcinoma. In 1992, Ro *et al* proposed the histological criteria of carcinosarcoma (2): (i) the concurrent presence of malignant epithelial and spindle cell components, between which there are transitional areas, and (ii) the sarcomatoid component expresses an epithelial phenotype. In this study, no transitional area was observed between the sarcomatous and carcinomatous components, but irregular intermingling was identified. Carcinosarcoma most commonly occurs in middle-aged and elderly men with a history of smoking or drinking. In the present case, the patient had a long history of smoking. Carcinosarcoma has been found in such diverse locations as the uterus, breast, thyroid, lung and upper gastrointestinal system (3). It is most frequently observed in the esophagus, while localization in the stomach has been less frequently identified (4,5). More than 80% of carcinosarcomas are located in the middle and/or lower esophagus. Macroscopically, the majority of carcinosarcomas are of the polypoid type and others are of the ulcerative type (6,7). Gastric carcinosarcoma typically presents with an elevated lesion or increased thickness of the gastric wall (8,9), and rarely presents with an ulcerated lesion (10). In the current case, a bulky pedunculated polypoid lesion in the middle of the esophagus and a huge discoid lesion in the lesser curvature with increased thickness of the gastric wall were observed. MCS of the esophagus and stomach has not been previously reported.

Immunocytochemistry is the gold standard for the diagnosis of carcinosarcoma, as upper gastrointestinal series (barium swallow), CT and even endoscopy are observed to be less efficient and accurate. It has been demonstrated that CEA, EMA, pancreatin, chromogranin A, CD56 and synaptophysin staining are highly specific markers for the carcinomatous components, while desmin, vimentin and smooth muscle/sarcomeric actin show affinity for the sarcomatous elements (11,12). In the present case, the immunohistochemical staining findings in both the esophageal and gastric lesions were consistent with the diagnosis of carcinosarcoma.

The histological origin of carcinosarcoma is debated, and two main hypotheses have been proposed. The first hypothesis is a stem cell theory of origin, with tumor stem cells differentiating toward epithelial neoplasm and mesenchymal metaplasia (13). The second hypothesis is a tumor collision theory, with neoplasm derived from the collision of two distinct neoplasms that are epithelial and mesenchymal in origin (14,15). Molecular analysis has revealed that the two components of carcinosarcoma have different genetic mutations, mainly involving the P53, cyclin D1, P16, MDM2 and CDK4 genes (16–21). P53 gene mutations exist in both the sarcomatous and carcinomatous components, but the type of mutation differs (16). Cyclin D1 gene amplification is frequently amplified in carcinosarcoma, particularly in the sarcomatous component (17). It has been demonstrated in the esophagus that the two components exhibited cyclin D1 gene amplification and p16 homozygous deletion, by differential polymerase chain reaction and fluorescence *in situ* hybridization (19). Certain studies have demonstrated that MDM2 and CDK4 were strongly implicated in the pathogenesis of carcinoma and sarcoma (14,20,21). CDK4 overexpression was observed in laryngeal squamous cell carcinoma, which was significantly correlated with tumor size and an advanced stage (21). Nikitakis *et al*(14) compared the expression of MDM2 and CDK4 in two cases of esophageal carcinosarcoma, and selected cases of esophageal squamous cell carcinoma with a prominent stromal reaction. The results supported the common epithelial origin of carcinosarcoma.

We assumed that carcinosarcoma is a type of malignant disease that is different from cancer or sarcoma, with its own unique pathological features. The diversity, complexity and mixed type of the two components reveal the possibility of origination from the same original proto-stem cells of carcinosarcomas. Under certain carcinogens, these proto-stem cells may commence pathological differentiation in different ways and present the traits of mutagenized malignant cells. In this case, the patient exhibited MCS of the esophagus and stomach. We suggest that it is possible that the tumor originated from the same original proto-stem cells. Further genetic analyses of the lesions of the two locations may assist in determining the cell source of carcinosarcoma.

With respect to the treatment of upper gastrointestinal carcinosarcoma, there is no recommended clinical management at present. The treatment modalities for esophageal carcinosarcoma include esophagectomy, endoscopic resection and chemo-radiotherapy (22,23). Esophagectomy has been traditionally considered as the first option for esophageal carcinosarcoma (24), but endoscopic therapy may represent an alternative to surgery for superficial carcinosarcoma. In gastric carcinosarcoma, the main therapy conducted is radical and comprises partial or total gastrectomy (10,25). At present, surgery is regarded to be the only curative treatment for gastric carcinosarcoma. Endoscopic polypectomy is a novel alternative to surgery for patients with polypoid carcinosarcoma confined to the mucosal layer with no involvement of the lymph nodes. Pesenti *et al*(23) conducted the first EMR for esophageal carcinosarcoma with good tolerance and a favorable prognosis. In the present case, with two lesions in both the esophagus and stomach, the patient was unable to tolerate radical esophagectomy or gastrectomy at the same time due to his poor condition. To improve the symptoms of esophageal obstruction and guarantee normal eating, esophageal EMR (palliative surgery) was performed and a series of treatments, including acid suppression, anti-inflammatory and hemostasis, followed. The patient demonstrated a good postoperative recovery without bleeding or perforation; however, the patient succumbed after seven months. We propose that if the patient had received laparoscopic gastric partial gastrectomy at the same time, he may have had a longer survival time and an improved prognosis. However, for age reasons, the patient refused to undergo further laparoscopic surgery.

In conclusion, carcinosarcoma is a rare tumor with limited clinical recognition and an extremely high rate of mis diagnosis. There are no reports regarding the occurrence of MCS in both the esophagus and stomach. Therefore, due to the polypoid non-invasive lesions in the esophagus, and the discoid non-invasive mass with an off-white surface in the stomach, extensive samples should be obtained for immunohistochemistry or genetic analysis to achieve a definite diagnosis. Immunocytochemistry has been the gold standard for the diagnosis of carcinosarcoma. Chemotherapy for carcinosarcoma was demonstrated to be ineffective. Compared with traditional surgery, therapeutic endoscopy has been proven to be less invasive; notably, it enables the esophagus to be preserved. Therefore, therapeutic endoscopy may provide an alternative method of treating carcinosarcoma, particularly MCS.

## Abbreviations:

CDK4: cyclin-dependent kinase 4
CK: cytokeratin
CT: computed tomography
EGD: esophagogastroduodenoscopy
EMA: epithelial membrane antigen
EMR: endoscopic mucosal resection
MCS: multiple carcinosarcomas
MDM2: murine double minute
NSE: neuron specific enolase
P53: protein 53
SMA: smooth muscle actin

## References

[1] Virchow RLK (1865). Vorlesungen uber Pathologie die krankhaften Geschwulste.

[2] Ro JY, Chen JL, Lee JS (1992). Sarcomatoid carcinoma of the lung: Immunohistochemaical and ultrastructural studies of 14 cases. Cancer.

[3] Robey-Cafferty SS, Gringon DJ, Ro JY (1990). Sarcomatoid carcinoma of the stomach: A report of three cases with immunohistochemical and ultrastructural observations. Cancer.

[4] Kanamoto A, Nakanishi Y, Ochiai A (2000). A case of small polypoid esophageal carcinoma with multidirectional differentiation, including neuroendocrine, squamous, ciliated glandular, and sarcomatous components. Arch Pathol Lab Med.

[5] Yamazaki K (2003). A gastric carcinosarcoma with neuroendocrine cell differentiation and undifferentiated spindle-shaped sarcoma component possibly progressing from the conventional tubular adenocarcinoma; an immunohistochemical and ultrastructural study. Virchows Arch.

[6] Iyomasa S, Kato H, Tachimori Y (1990). Carcinosarcoma of the esophagus: a twenty-case study. Jpn J Clin Oncol.

[7] Kuo CJ, Lin TN, Lin CJ (2010). Clinical manifestation of esophageal carcinosarcoma: a Taiwan experience. Dis Esophagus.

[8] Ashida K, Wamata T, Sugesawa A (1998). A case of so-called carcinosarcoma of the stomach. J Jpn Surg Assoc.

[9] Tanimura H, Furuta M (1967). Carcinosarcoma of the stomach. Am J Surg.

[10] Kikuyama R, Tanaka K, Tano S (2009). A case of gastric carcinosarcoma. Endoscopy.

[11] Teramachi K, Kanomata N, Hasebe T (2003). Carcinosarcoma (pure endocrine cell carcinoma with sarcoma components) of the stomach. Pathol Int.

[12] Sato Y, Shimozono T, Kawano S (2001). Gastric carcinosarcoma, coexistence of adenosquamous carcinoma and rhabdomyosarcoma: a case report. Histopathology.

[13] Ota S, Kato A, Kobayashi H (1998). Monoclonal origin of an esophageal carcinosarcoma producing granulocyte-colony stimulating factor: a case report. Cancer.

[14] Nikitakis NG, Drachenberg CB, Papadimitriou JC (2002). MDM2 and CDK4 expression in carcinosarcoma of the esophagus: comparison with squamous cell carcinoma and review of the literature. Exp Mol Pathol.

[15] Meyer R (1984). Current findings for the interpretation of ‘tight junctions’ as lipid structures. Acta Histochem Suppl.

[16] Nakagawa S, Nishimaki T, Suzuki T (1999). Histogenetic heterogeneity in carcinosarcoma of the esophagus: report of a case with immunohistochemical and molecular analyses. Dig Dis Sci.

[17] Suzuki H, Moriya J, Nakahata A (1998). Cyclin D1 gene amplification in esophageal carcinosarcoma shown by differential polymerase chain reaction. Hum Pathol.

[18] Iwaya T, Maesawa C, Tamura G (1997). Esophageal carcinosarcoma: a genetic analysis. Gastroenterology.

[19] Suzuki H, Fujioka Y, Nagashima K (1998). Cyclin D1 gene amplification and p16 gene deletion in patients with esophageal carcinosarcoma. Diagn Mol Pathol.

[20] Momand J, Jung D, Wilczynski S (1998). The MDM2 gene amplification database. Nucleic Acids Res.

[21] Dong Y, Sui L, Sugimoto K (2001). Cyclin D1-CDK4 Complex: a possible critical factor for cell proliferation and prognosis in laryngeal squamous cell carcinomas. Int J Cancer.

[22] Hung JJ, Li AF, Liu JS, Lin YS, Hsu WH (2008). Esophageal carcinosarcoma with basaloid squamous cell carcinoma and osteosarcoma. Ann Thorac Surg.

[23] Pesenti C, Bories E, Danisi C (2004). Endoscopic treatment of esophageal carcinosarcoma: report of a case. Endoscopy.

[24] Ziauddin MF, Rodriguez HE, Quiros ED, Connolly MM, Podbielski FJ (2001). Carcinosarcoma of the esophagus-pattern of recurrence. Dig Surg.

[25] Ikeda Y, Kosugi S, Nishikura K (2007). Gastric carcinosarcoma presenting as a huge epigastric mass. Gastric Cancer.

